# Hématome sous capsulaire de foie compliquant une pré-éclampsie: à propos de 6 cas

**DOI:** 10.4314/pamj.v9i1.71229

**Published:** 2011-08-28

**Authors:** Nisrine Mamouni, Ali Derkaoui, Hakima Bougern, Chehrazad Bouchikhi, Hikmat Chaara, Abdelaziz Banani, Melhouf Moulay Abdelilah

**Affiliations:** 1 CHU Hassan II, Fès, Maroc

**Keywords:** Hématome, Sous capsulaire, Foie, Pré-éclampsie, HELLP Syndrome, Maroc

## Abstract

L'hématome sous capsulaire du foie (HSCF) est une complication rare mais gravissime de la grossesse. Devant une symptomatologie clinique souvent non spécifique et un tableau biologique retardé, son diagnostic est basé essentiellement sur les moyens de l'imagerie (échographie, TDM, IRM). Son traitement est fonction de l'intégrité ou non de la capsule de Glisson. Nous rapportons les observations de 6 patientes, à travers une étude rétrospective s’étalant sur la période du Janvier 2005 à Octobre 2008, incluant tous les cas de preeclampsie colligés au service de gynécologie obstétrique du CHU Hassan II. Durant la période d’étude, L'incidence de l'hématome sous capsulaire de foie chez les patientes préeclamptiques admises durant la période d’étude est de 1,49 %. Aucune des patientes n'a benificié d'un suivi prénatal au sein de notre formation. La moyenne d’âge des patientes est de 37,6 ans avec des extrêmes allant de 33 à 45 ans. La gestité moyenne était de 4,8 avec une parité moyenne de 4,5.l'hematome sous capsulaire est survenu en post partum chez tous nos cas avec un délai moyen de 4 jours et des extrêmes allant de J0 et J10 du post partum .Toutes les patientes ont présenté un HELLP syndrome concomitant à la survenue de cette complication gravissime.Le diagnostic positif s'est basé sur les données échographiques dans 5 cas (hemoperitoine –HSCF).l’équipe a opté pour une abstention thérapeutique avec surveillance armée chez 2 cas et l'exploration chirurgicale a été indiquée chez quatre patientes en instabilité hemodynamique.Nous avons déploré deux cas de décès maternel.

## Introduction

L'hématome sous capsulaire du foie (HSF) est une complication rare et gravissime au cours de la prééclampsie survenant dans un tableau clinique relativement stéréotypé, mais non spécifique, ce qui souvent entraîne un retard du diagnostic. Sa rupture secondaire est l'une des plus graves complications obstétricales avec une mortalité maternelle estimée à 50 à 75 % et une mortalité fœtale à 60 à 80 % [1]. Nous proposons à partir de six cas cliniques de faire le point sur les aspects epidemiologiques, cliniques, paracliniques, et thérapeutiques de l'hématome sous capsulaire (HSC) du foie survenant dans le cadre d'une prééclampsie.

## Cas Clinique

### Cas 1

Mme FB, âgée de 33 ans, sans antécédent pathologique notable, multipare, admise pour prise en charge d'une hypertension artérielle sévère sur une grossesse de 35 semaines (SA). A l'admission, la patiente a été consciente avec une pression artérielle systolique à 240 mmHg et une diastolique à 120 mmHg. La protéinurie a été positive. Le bilan biologique initial a été normal. L'extraction est réalisée par voie haute pour sauvetage maternel ayant donné naissance à un nouveau né mature avec un Apgar à 10. En post opératoire immédiat la patiente a présenté une instabilité hémodynamique avec un ballonnement abdominal .Une laparotomie exploratrice réalisée a mis en évidence un hémoperitone de grande abondance suite à la rupture d'un hématome sous capsulaire du foie situé sur la face inférieure. La patiente a benificié d'un lavage péritonéal et mise en place d'un packing. Le décès maternel est survenu en post opératoire dans un tableau d’état de choc hémorragique réfractaire.

### Cas 2

Mme FK, âgée de 34 ans, sans antécédent pathologique notable, primigeste, admise pour prise en charge d'une éclampsie sur grossesse de 26 SA. A son admission, la patiente avait un GCS à 13 .la pression systolique à 170 mmHg et la pression diastolique à110 mmHg avec une protéinurie positive. Le bilan biologique a été normal .un traitement conservateur a été décidé et la patiente a été mise sous la nicardipine à la seringue autopousseuse associée au sulfate de magnésium .Après 24 heures de l'admission, une césarienne a été réalisée vue la perturbation du bilan biologique (un HELLP syndrome complet) donnant naissance à un mort né. La patiente a présenté une instabilité hémodynamique à 36 heures du geste chirurgical. L’échographie a montré la présence d'un épanchement intraperitoneal de grande abondance. Une laparotomie a été réalisée permettant l’évacuation de l’épanchement et mise en place d'un packing en inter hépato- diaphragmatique. La patiente est décédée après 24 heures plutard dans un tableau d’état de choc hémorragique et défaillance multiviscérale.

### Cas 3

Mme AF, âgée de 45 ans, multipare, admise à une semaine après un accouchement par voie basse dans un tableau de choc hémorragique. La grossesse est non suivie, l′accouchement s′est déroulé à domicile donnant naissance à un mort-né. La patiente a rapporté la survenue de douleurs intenses de l′hypochondre droit la veille de son admission n′ayant pas motivé sa consultation. La patiente a été admise en état de choc hémodynamique. La protéinurie a été positive. L'abdomen a été sensible de façon diffuse avec un maximum au niveau de l'hypochondre droit. L′examen gynécologique a montré un col fermé, un utérus augmenté de taille sans saignement vaginal. Le bilan biologique a objectivé un HELLP syndrome complet. Une échographie abdominale réalisée en urgence a montré un épanchement péritonéal de grande abondance.une laparotomie exploratrice a été décidée, avec l'aspiration d'un hémopéritoine de 2000cc permettant la découverte d'un hématome sous capsulaire localisé sur la face diaphragmatique du foie de 4 centimètres de diamètre fissuré avec mise en place d'un packing .Les suites opératoires ont été marquées par l′amélioration progressive du bilan biologique. La patiente est sortie de l'hôpital au 15^e^ jour sans séquelles.

### Cas 4

Mme RM, âgée de 36 ans, paucipare, admise après dix jours d'un accouchement par voie basse à domicile dans un tableau d'abdomen aigu. La grossesse estimée à terme n'a pas été suivie. L'accouchement a donné naissance à un nouveau né ayant une bonne adaptation à la vie extrauterine. L′examen à son admission trouve une patiente consciente ayant une pression artérielle systolique à 140 mmHg. La protéinurie a été positive. L′abdomen est ballonné et sensible au niveau de l'hypochondre droit, sans saignement par voie basse. Le bilan biologique a révélé un HELLP complet. L′échographie abdominale a montré un hémopéritoine de moyenne abondance et un hématome sous capsulaire du foie intéressant le lobe gauche faisant 10 x 3,6 cm. Devant la stabilité hémodynamique, la décision a été de surveiller la patiente en milieu de réanimation, sous traitement symptomatique à base de remplissage vasculaire par les solutés cristalloïdes et de transfusion par des concentrés de globules rouges. La pression artérielle s'est stabilisée par la nicardipine (Loxen inj) à la seringue électrique. L′évolution a été marquée par l'amélioration du bilan biologique (la régression de la cytolyse et de la thrombopénie en 10 jours) et la régression progressive de l′hématome sous capsulaire et de l′hémopéritoine. La patiente est sortie de l′hôpital après quinze jours. L′échographie abdominale de contrôle réalisée après 3 mois a montré la régression complète de l′hématome sous capsulaire.

### Cas 5

Mme NB, âgée de 40 ans, multipare, admise pour suspicion de péritonite aigue généralisée à une semaine du post-partum .la patiente ayant accouché à domicile donnant naissance à un nouveau né de 2500g avec bonne adaptation à la vie extrauterine. Une semaine après l′accouchement, La patiente a présenté des douleurs abdominales diffuses associées à des vomissements alimentaires puis bilieux. L′examen clinique trouve une patiente asthénique, fébrile à 38° ;C, sa pression artérielle systolique à 90 mmHg avec un ventre de bois et une protéinurie positive.Le bilan biologique a objectivé une cytolyse hépatique avec une hyperleucocytose (24600 /mm^3^). L′échographie abdominale a mis en évidence un épanchement intraperitoneal de moyenne abondance et un utérus vide post-gravidique augmenté de taille.L′exploration chirurgicale a objectivé la présence d'un hémopéritoine de 800 millilitres évacué avec la présence d'un hématome sous capsulaire du lobe hépatique droit rompu d'o[ugrave] la réalisation de points de suture hépatiques d'hémostase. Trois semaines après l′intervention chirurgicale, l’échographie de contrôle a montré la résorption de l'hématome. La patiente a été déclarée sortante après dix-sept jours d'hospitalisation.

### Cas 6

**Figure 1:**
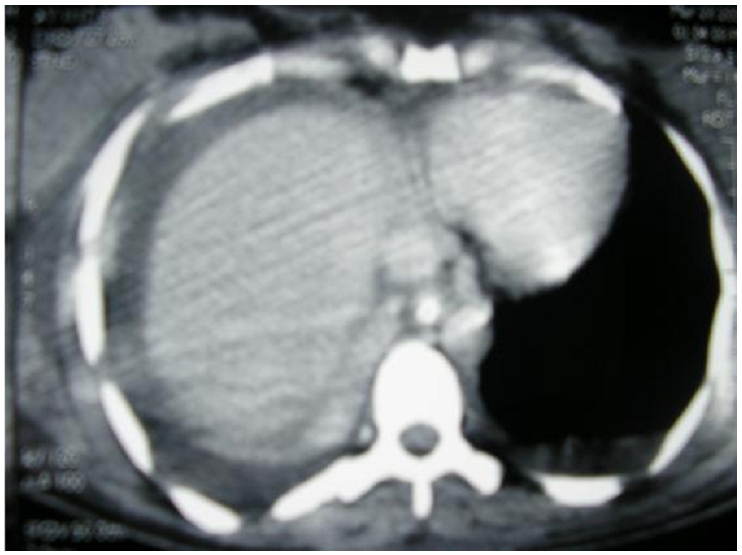
Scanner abdominale montrant la présence d'un hématome sous capsulaire de foie

**Figure 2:**
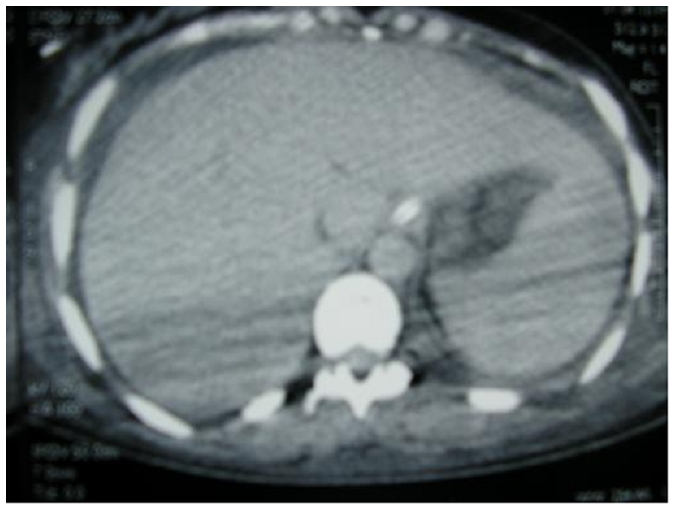
Scanner abdominale montrant un hématome sous capsulaire du foie et inter splénique

**Figure 3:**
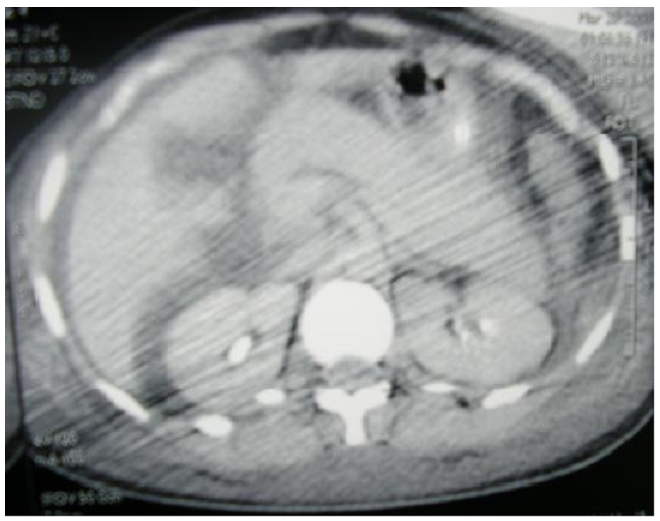
Scanner abdominale montrant un hématome sous capsulaire du foie et en péri-rénale droit

Mme KK, âgée de 38 ans, paucipare, admise pour une éclampsie sur grossesse à terme. A son admission, la patiente a été comateuse avec une pression artérielle systolique à 180 mmHg. L'extraction fœtale urgente s'est déroulée par voie haute donnant naissance à un nouveau-né Apgar à 8. Au premier jour du post partum, la patiente a développé un ictère cutaneomuqueux avec une sensibilité de l'hypochondre droit.le bilan biologique de contrôle objectivant une anémie normochrome à 5g/dl, une bilirubinémie totale à 160 mg/l et une cytolyse hépatique marquée. L’échographie abdominale réalisée objectivant un hématome sous capsulaire de foie de 6 cm de grand axe associé à un épanchement intraperitoneal de moyenne abondance. La tomodensitométrie abdominale réalisée confirmant les données échographiques. L'abstention thérapeutique a été décidée vu la stabilité hémodynamique avec mise en place des mesures de réanimation strictes et transfusion par des concentrés globulaires de plasma congelé et de culots plaquettaires.la patiente est restée stable sur le plan hémodynamique avec amélioration des paramètres biologiques et fut déclarée sortante après 14 jours d'hospitalisation. (Figures 1, 2, 3)

Les caractéristiques cliniques et paracliniques de nos patientes sont résumées dans les Tableau 1 et 2.

**Tableau 1:** Tableau comparatif des paramètres cliniques de 6 patientes traitées au CHU Hassan II de Fès (Maroc) pour hématome sous capsulaire du foie compliquant une pré-éclampsie

	Cas 1	Cas 2	Cas 3	Cas 4	Cas 5	Cas 6	moyenne
							
Age	33	34	45	36	40	38	37,6
Motif d'admission	Etat de choc	éclampsie	Etat de choc	Etat de choc	Etat de choc	Eclampsie	_
Antécédent	_	_	_	_	_	MFIU	_
Gestité	3	1	8	4	10	3	4,8
Parité	2	0	8	4	10	3	4,5
Age gestationnel (SA)	35	25	37	37	38	37	_
GCS	15	10	15	15	15	8	13
PAS	240	170	70	140	90	180	148,3
PAD	120	110	30	100	60	120	90
PROTEINURIE							
(B.reactive)	++++	+++	++++	++++	++++	++++	_
OMI	+++	+++	++	+++	++	+++	_

OMI: œdèmes des membres inférieurs; GCS: Score de Glasgow; PAS: pression artérielle systolique; PAD: pression artérielle diastolique; MFIU: malformation Intra-Utérine

**Tableau 2:** les paramètres para cliniques et évolutifs de 6 patientes traitées au CHU Hassan II de Fès (Maroc) pour hématome sous capsulaire du foie compliquant une pré-éclampsie

	Cas1	Cas 2	Cas3	Cas 4	Cas 5	Cas 6	Moyenne
							
Hémoglobine au diagnostic (g/dl)	2,4	6	7,2	8	9	5	7,4
Taux plaquettes au diagnostic (mm3)	25000	55000	80000	100000	105000	46000	67666
GOT/GPT (UI/l)	120	520	350	323	120	260	282,1
Taux de Prothrombine(%)	10	40	45	60	55	70	46,6
Creatininemie (mg/l )	9	12,9	103	8,5	30	11,8	29,2
Echographie abdominale	+	+	+	+	+	+	
TDM abdominale	_	_	_	_	_	_	
Mortalité maternelle	Décès	Décès	Non	Non	Non	Non	
Mortalité fœtale	Vivant	Mort-né	Mort-né	Vivant	Vivant	Vivant	

## Discussion

L'hématome sous capsulaire du foie est une complication rare de la grossesse dont l'incidence de survenue est estimée à1 pour 45000 naissances à 1 pour 250 000 naissances) survenant le plus souvent chez la multipare âgée de plus de 30 ans [1]. L'hématome sous capsulaire du foie se voit dans 50 % des cas après 36 semaines d'aménorrhée, se révèle avant le travail chez 85 % des cas et dans le post-partum immédiat chez 15 % des cas [1]. Dans notre série, L'hématome sous capsulaire de foie est survenu en post partum chez tous nos cas, avec un délai moyen de survenue au cinquième jour du post-partum (des extrêmes : J0- J10). En dehors de la preeclampsie, l'HSF peut survenir lors d'un traumatisme direct ou sur une lésion focale hépatique préexistante (hémangiome, adénome, hyperplasie nodulaire focale) [2].

Sur le plan physiopathologique, deux théories sont avancées.La première affirme que l'atteinte hépatique prédomine dans la zone periportale, comprenant de nombreux dépôts obstructifs de fibrine disséminés dans les sinusoïdes hépatiques, une nécrose hepathocytaire focale à l'origine de la cytolyse hépatique, des thromboses et des hémorragies intra hépatiques. L'ensemble de ses lésions participe à la congestion sinusoïdale avec hyperpression intra parenchymateuse qui peut être responsable d'hématome sous capsulaire du foie et d'hémopéritoine [3].

La deuxième théorie affirme que l'ischémie utéro-placentaire est responsable du spasme des capillaires portes par le biais des substances vasoactives qu'elle libère, il s'en suit l'ischémie et la nécrose du foie. Au cours du HELLP, les lésions d'infarcissement hépatique s'intègrent dans un processus systémique consécutif au développement d'une coagulation intra vasculaire disséminée. La traduction histologique de ces phénomènes est représentée par la présence de foyers de nécrose et d'hémorragie hepathocytaire et periportale.

Sur le plan clinique, on peut distinguer le tableau de l'HSF non rompu qui correspond à un tableau de prééclampsie plus ou moins sévère, associé à un syndrome douloureux abdominal épigastrique et/ ou de l'hypochondre droit. Il s'y associe souvent des signes peu spécifiques tels que des céphalées, des nausées voire des vomissements qui peuvent retarder le diagnostic [1].

L'hématome rompu réalise un tableau d'urgence abdominale avec aggravation du syndrome douloureux abdominal et l'installation d'un état de choc hypovolémique. Malgré son aspect stéréotypé, la non-spécificité du tableau clinique entraîne souvent un retard diagnostique préjudiciable aux patientes [4]. Ce diagnostic doit être évoqué tout au long de la grossesse et dans la période du post-partum, et au moindre doute la réalisation d'un bilan radiologique (échographie, scanner) s'avère nécessaire.Dans notre série, la rupture de l'hématome est survenue chez 5 cas se manifestant par un tableau de choc hypovolemique réfractaire.

En l'absence d’état de choc hémorragique, le diagnostic est essentiellement échographique par visualisation d'un hématome sous capsulaire ou d'une hyperéchogénicité de la capsule de Glisson qui peut témoigner d'un début de décollement. L'examen échographique contribue au dépistage des complications hépatiques chez les femmes présentant une cytolyse hépatique dans le cadre du HELLP, pose le diagnostic de HSCF chez les patientes stables, et permet le suivi de ces patientes après abstention thérapeutique.

La tomodensitométrie reste l'examen de choix pour l'exploration de cette pathologie. C'est la technique la plus sensible pour la mise en évidence de l'ischémie hépatique, pour la recherche de signe de complications hémorragiques (hémorragie intaparenchymateuse, l'hématome sous capsulaire et l'hémopéritoine), ainsi que l’établissement du diagnostic différentiel.

L'imagerie par résonance magnétique, actuellement en cours d’évaluation, aura vraisemblablement dans l'avenir sa place dans ce type de pathologie, d'autant plus qu'elle n'est pas irradiante. Elle n'a pas d'intérêt dans un contexte d'urgence, mais peut s'avérer utile dans la détection secondaire d’éventuelles lésions causales. Sa réalisation ne doit pas retarder l'action thérapeutique [5].

Barton et al [6] ont étudié l'intérêt de l'imagerie hépatique dans le contexte du HELLP syndrome. Trente-quatre patientes présentant un HELLP syndrome ont été incluses. Trente trois patientes ont eu une tomodensitométrie (TDM), quatre d'entre elles ont bénéficié d'une imagerie par résonance magnétique (IRM) et cinq patientes une échographie. Toutes les patientes présentaient cliniquement une douleur de l'hypochondre droit. Dans 45 % des cas, l'examen radiologique était anormal montrant un hématome sous capsulaire du foie ou une hémorragie intra parenchymateuse.Dans notre série l’échographie abdominale a été réalisée chez 9 cas parmi les patientes préeclamptiques admises pendant la période d’étude ayant présenté des douleurs épigastriques et /ou cytolyse hépatique ayant objectivé un hémopéritoine dans 6 cas et un HSCF dans 2 cas confirmé par la tomodensitométrie abdominale dans un cas.

L'attitude thérapeutique de l'HSCF doit prendre en compte l’état hémodynamique de la patiente et l'intégrité ou non de la capsule de Glisson. La réanimation pré, per et postopératoire, vise à corriger l'hypertension artérielle, l'hypovolémie et les altérations de la coagulation.Le premier temps opératoire est toujours l'extraction fœtale par césarienne.Seule celle-ci permet de stopper l’évolution des lésions hépatiques liées à la pré-éclampsie et au HELLP syndrome.

En l'absence de rupture de la capsule hépatique, une surveillance étroite et un traitement symptomatique par la correction des troubles de la coagulation peut être envisagé.En cas de rupture de la capsule de Glisson, l'attitude thérapeutique dominante consiste en une prise en charge chirurgicale conservatrice par mise en place d'un packing du foie [5, 7, 8]. Depuis, d'autres alternatives thérapeutiques ont été évaluées, elles reposent le plus souvent sur des séries rétrospectives regroupant un petit nombre de patientes. Dans ce cadre, l'intérêt de l'interruption artérielle hépatique (par embolisation ou ligature chirurgicale) a été évalué par une étude rétrospective portant sur huit patientes [9]. Pour deux patientes l'embolisation a été réalisée après l’échec de l'hémostase chirurgicale locale. Un arrêt du saignement a été obtenu pour six parturientes, les auteurs soulignent le risque de gangrène vésiculaire et de nécrose hépatique liée à la technique de ligature chirurgicale.

Dans une autre série de 141 cas d'HSCF [10] , L'analyse en fonction de l'attitude thérapeutique montre que c'est avec le packing ou l'embolisation hépatique sélective que l'on obtient la meilleure survie maternelle, respectivement 80 et 90 % [10]. Les autres techniques, telles la ligature chirurgicale des artères hépatiques ou la résection des plages de nécrose hépatique sont associées à une mortalité maternelle importante supérieure à 30 % [10].

Dans les six cas rapportés dans notre série, la décision d′intervention était surtout basée sur l′état hémodynamique, chez quatre patientes, l′instabilité hémodynamique et les données de l′échographie nous ont orientées vers une laparotomie exploratrice en urgence. Dans deux cas, la stabilité de l′état hémodynamique était en faveur de l′abstention chirurgicale avec surveillance et réanimation intensives. Enfin, l'administration de facteur VIIa recombinant seul en l'absence de rupture hépatique ou associée à la chirurgie a été rapportée dans la littérature [11]. En cas de défaillance hépatique aiguë, un recours à la transplantation peut être envisagé.

La survenue d'un hématome sous capsulaire du foie rompu est responsable d′une mortalité materno-fœtale élevée avec 18 % de mortalité maternelle selon Smith et al. [2], 56 à 75 % de mortalité maternelle et 60 à 70 % de mortalité fœtale selon Pezet et al [3]. Dans notre série, la mortalité maternelle et fœtale est estimée au tiers des cas. La prévention est essentielle dans notre contexte qui doit être basée sur le suivi des grossesses, la médicalisation des accouchements, l’éducation de la population et le dépistage des grossesses compliquées de pré-éclampsie, chose qui va permettre certainement de réduire la mortalité materno-fœtale liée à cette pathologie.

## Conclusion

L'hématome sous capsulaire du foie est une complication rare et grave responsable d'une mortalité materno-fœtale élevée. L'amélioration du pronostic passe par la surveillance et le traitement efficace de la pré-éclampsie. L'exploration radiologique (échographie, scanner) doit être d'indication large dans le cadre de la pré-éclampsie. L'attitude thérapeutique en l'absence de consensus doit tenir compte de l'état hémodynamique et de l'état de la capsule de Glisson. L'abstention chirurgicale doit s'accompagner d'une réanimation adéquate et d'une surveillance clinique, biologique et radiologique.
